# Significance of the urokinase-type plasminogen activator and its receptor in the progression of focal segmental glomerulosclerosis in clinical and mouse models

**DOI:** 10.1186/s12929-016-0242-7

**Published:** 2016-02-04

**Authors:** Jin-Shuen Chen, Li-Chien Chang, Chung-Ze Wu, Tzu-Ling Tseng, Jui-An Lin, Yuh-Feng Lin, Chao-Wen Cheng

**Affiliations:** Division of Nephrology, Department of Internal Medicine, Tri-Service General Hospital, National Defense Medical Center, No. 325, Section 2, Chenggong Road, Neihu District, Taipei, 114 Taiwan; School of Pharmacy, National Defense Medical Center, Taipei, Taiwan; Department of Internal Medicine, Shuang Ho Hospital, Taipei Medical University, New Taipei City, Taiwan; Biomedical Technology & Device Research Laboratories, Industrial Technology Research Institute, Hsinchu, Taiwan; Graduate Institute of Clinical Medicine, College of Medicine, Taipei Medical University, No. 250, Wu-Xing Street, Taipei, 110 Taiwan; Graduate Institute of Medical Sciences, National Defense Medical Center, Taipei, Taiwan

**Keywords:** Focal and segmental glomerulosclerosis, Soluble urokinase-type plasminogen activator receptor, Urokinase-type plasminogen activator, Th1/Th2 balance

## Abstract

**Background:**

suPAR biomarker generally considered a pathogenic factor in FSGS. However, studies have been published that dispute this conclusion. The current study was designed to investigate the roles of uPA and suPAR in FSGS in clinical and mouse models.

**Methods:**

Clinical subjects including those with biopsy-proven FSGS and MCD were enrolled. To verify the role of uPA in FSGS, Adriamycin was used to induce FSGS in uPA knockout (uPA^−/−^) and BALB/c (WT) mice. Proteinuria and suPAR, the cleaved/intact forms of the circulating suPAR, and possible proteases involving cleavage of the suPAR were also studied.

**Results:**

FSGS clinical cases presented significantly higher serum levels of suPAR and Cr and lower serum levels of uPA. In the mice model, the uPA^−/−^ group exhibited faster disease progression and worsening proteinuria than the WT group. In addition, the uPA^−/−^ group had higher plasma suPAR levels, glomerular cell apoptosis, and dysregulation of the Th1/Th2 balance. In an analysis of suPAR variants in FSGS, both the intact and cleaved forms of the suPAR were higher in clinical subjects and the mouse model. However, the process of suPAR cleavage was not mediated by enzymatic activities of the uPA, elastase, or cathepsin G.

**Conclusions:**

A deficiency of uPA accelerated the progression of Adriamycin-induced mouse FSGS model. Decrease of serum uPA levels may be an indicator of the progression of FSGS in clinical subjects and animal models.

## Background

Focal and segmental glomerulosclerosis (FSGS) accounts for up to 20 % of glomerular disease and is a major cause of end-stage renal disease (ESRD). The pathogenesis of FSGS and underlying causes remain unclear; however, it was postulated that certain circulating factors may be responsible for initiating renal injury [1]. In 2011, Wei et al. identified increased levels of the soluble urokinase-type plasminogen activator (uPA) receptor (suPAR) in approximately two-thirds of patients with FSGS, and suPAR levels were predictive of FSGS recurrence in transplanted kidneys [2]. They suggested that serum suPAR may be a circulating factor causing FSGS [3]. In contrast, a cohort study by Meijers et al. did not support use of the suPAR as a biomarker for FSGS [4], and suggested that the currently available measurement by suPAR assays had no proven clinical value in patients with FSGS [5]. The role of the suPAR in the progression of FSGS remains controversial.

The suPAR is derived from the uPAR, which is expressed on the cell surface of multiple cell types, including vascular endothelial cells and immune cells. Cell-surface plasminogen activation is catalyzed by the binding of the uPA and uPAR, which facilitates the binding of plasminogen to the cell surface, and controls fibrinolysis [6, 7]. In addition, the uPAR is also involved in many other non-proteolytic biological processes, such as cell migration, adhesion, angiogenesis, and cell proliferation [8–10]. The primary structure of the mature uPAR is formed by approximately 90-amino-acid, cysteine-rich LY6-like, short linker regions connecting three homologous domains (D1, D2, and D3, numbered from the N-terminus) that are linked to cell membranes by glycosylphosphatidylinositol (GPI) [11, 12]. The uPAR binds the uPA in a pocket comprising three extracellular domains, while the entire external surface interacts with other signaling molecules such as integrins, formyl peptide receptors, and epithelial growth factor receptor, the mannose-6-phosphate receptor, the family of low-density lipoproteins receptor-related proteins, p130, and others [13–15]. The GPI-anchored membranous uPAR can be cleaved to various soluble forms of the uPAR (suPAR), including glycolipid-anchored and soluble forms of the intact uPAR D1 ~ D3, and the cleaved receptor, uPAR (D2 ~ D3) and uPAR (D1) [16, 17]. It was suggested that the proteolytic cleavage of the uPAR between D1 and D2 depends on the binding of the uPA and plasmin [18]. suPAR levels in human body fluids are quite stable in healthy individuals, while increased levels can be observed in the plasma and serum with several disease conditions, such as immunological disorders and cancers [19–21]. Both intact and cleaved suPAR variants may have diagnostic and prognostic values in cancer, inflammation, and metabolic diseases; in addition, cleaved forms of the suPAR were suggested to be stronger prognostic markers for cancer diagnoses [22]. To the present, the composition of different circulating suPAR variants in FSGS patients has not been studied.

In general, it is thought that the suPAR scavenges the uPA and prevents its interaction with the membrane-anchored uPAR. However, since the circulating suPAR can cause FSGS by activating the podocyte β3 integrin downstream signaling pathway [2], there is an alternative hypothesis in which the uPA may also work as a scavenger for the suPAR in FSGS. The present study was designed to examine the role of the uPA in the pathogenesis of FSGS and further elucidate its relationship with suPAR levels.

## Methods

### Human subjects

Approval for the human subject study was granted (TSGHIRB: 094-05-0031) by the institutional review board of the Tri-service General Hospital (Taipei, Taiwan). Written consent was taken from all participants to collect and store the samples and to use medical outcome data for the purpose of the study. All participants were over 20 years old and capable of providing informed consent. Subjects seen in the Tri-service General Hospital Renal Clinic were included based on the results of a percutaneous renal biopsy with a diagnosis of FSGS and of minimal-change disease (MCD). Eighteen FSGS and 22 MCD patients were enrolled in this study. Urine and blood samples were obtained at the time of the renal biopsy. Samples were stored at − 80 °C before being analyzed.

### FSGS mice model

Healthy female Balb/c inbred mice (WT) 7 weeks of ages were obtained from the National Laboratory Animal Breeding and Research Center (Taipei, Taiwan). The uPA^−/−^ Balb/c mice (C.129S2-Plautm1Mlg/J) were originally purchased from Jackson Laboratory (Bar Harbor, Maine, USA), and bred in the animal center of National Defense Medical Center. After arrival, the animals were randomly allocated in groups of 6 in macrolon II cages. Animals were kept in a controlled specific pathogen-free environment, fed standard rodent chow *ad libitum*, under a 12 h day/ 12 h night rhythm. The animals were allowed to acclimatize for 1 week before starting the experimental protocol and were monitored daily for general health. Age (8 weeks) and body weight (~20 g) matched WT and uPA^−/−^ mice were intravenously treated with Adriamycin at a dose of 10 mg/kg. At weekly intervals (W0 ~ 4), indicated numbers of WT and uPA^−/−^ mice were euthanized (W0, *n* = 10; W1, *n* = 27; W2, *n* = 18; W3, *n* = 24; W4, *n* = 11). Blood samples were collected into a 1.5-ml microtube containing heparin through the retro-orbital venous plexus, and were then centrifuged (at 2500 rpm for 15 min), and the supernatant containing the plasma was withdrawn and stored at − 70 °C until for the following study. Spot urine samples were collected at indicated time point. The kidney tissues were obtained at the time of euthanization.

The animal experiments were conducted according to all applicable provisions of the Taiwan Animal Protection Act of 1998. This study was approved by the Animal Experimentation Ethical Committee of the National Defense Medical Center under permit number IACUC-13-017.

### Enzyme-linked immunosorbent assay (ELISA)

Human serum levels of the suPAR were determined using a Human uPAR Quantikine ELISA Kit (DUP00, R&D, Minneapolis, MN) according to the manufacturer’s protocol. Briefly, a capture antibody was pre-coated onto microplate wells and incubated overnight at room temperature (RT). Standards and samples were introduced to the wells and incubated for 2 h at RT. After washing, a biotinylated detection antibody was added to each well and incubated for 2 h at RT, followed by the addition of 100 μL streptavidin horseradish peroxidase (HRP) and incubation for 20 min at RT. A chromogen TMB substrate solution was added to the wells and incubated for 30 min at RT. Fifty microliters of stop solution was added to each well and read at 450 nm within 30 min. The method for the mouse plasma suPAR was performed according to the Mouse uPAR DuoSet (DY531, R&D). Ten individual samples at indicated time points were randomly selected for testing.

### Western blotting

Protein concentration in the human serum/mouse plasma samples was determined by the bicinchoninic acid (BCA) assay (Pierce, Rockford, IL). Equal amounts (30 μg) of protein from each samples were separated by sodium dodecylsulfate polyacrylamide gel electrophoresis (SDS-PAGE). The gel was equilibrated in transfer buffer at RT, and the protein was transferred onto polyvinylidene difluoride membranes (Millipore Immobilon-P, Sigma, St. Louis, MO) for 2.5 h at 4 °C in transfer buffer. The membranes were then blocked with 2 % bovine serum albumin (BSA) of TBST at 4 °C overnight. The next day, membranes were washed with TBST, and blots were individually incubated with antibodies against the human uPAR (MAB807, R&D) or mouse uPAR (MAB531, R&D) overnight at 4 °C, followed by incubation with a goat anti-rabbit antibodyfor 30 min at RT. Bindings were visualized with the Western Lightning Chemiluminescence Reagent *Plus* (Perkin-Elmer Life Sciences, Boston, MA) and exposed to Kodak film (Rochester, NY). Five individual samples at indicated time points were randomly selected for testing.

### Evaluation of renal histopathology

Formalin-fixed and paraffin-embedded kidney tissues were cut and stained with periodic acid-Schiff (PAS) stain and colloidal iron for the general histological examination as previously described [23]. Furthermore, to evaluate the severity of glomerular injury, glomeruli were examined using an Aperio digital microscope and quantified with the Scanscope digital program [24]. The developed tissues were counterstained with hematoxylin. Sections were then observed with an optical photomicroscope. Negative controls were performed by omitting the primary antibodies.

### Measurement of the helper T-cell 1 (Th1)/Th2 immune response

Mouse plasma concentrations of immunoglobulin G1 (IgG1), IgG2a, and IgG3 were measured using an ELISA as previously described [25]. IgG1, IgG2a, and IgG3 mouse reference sera (mouse IgG1, IgG2a, and IgG3 quantitation kits; Enzo Life Sciences, Farmingdale, NY) were used to construct a standard curve according to the manufacturer’s instructions. Ten individual samples at indicated time points were randomly selected for testing.

### Assay of cathepsin G and elastase activity

Elastase activity was detected as previously described [26], 50 μl blood plasma was incubated at 37 °C for 24 h with 50 μl of 1 mM elastase substrate (M4765, N-methoxy-succinyl-alanyl-alanyl-prolyl-valyl-p-nitroanilide, Sigma). The absorbance was measured on a microplate reader at 410 nm. The activity of cathepsin G was evaluated by a Cathepsin G Activity Assay Kit (ab126780, Abcam, Cambridge, MA), and all procedures were performed according to the manufacturer’s instructions. Six individual samples at indicated time points were randomly selected for testing.

### Statistical analysis

The statistical analysis of differences between groups was performed by a *t*-test. Significance was defined as *p* < 0.05. Pearson correlation coefficients were calculated for normally distributed variables; otherwise Spearman correlation coefficients were calculated. Data are expressed as the mean ± standard error (SE).

## Results

### FSGS patients showed higher suPAR and lower uPA levels

**Fig. 1:**
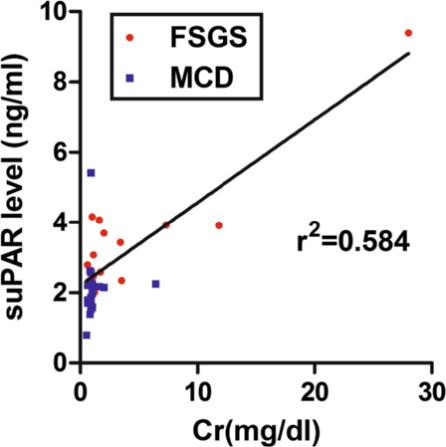
Moderate correlation between human plasma soluble urokinase-type plasminogen activator (uPA) receptor (suPAR) and serum creatinine in combination of FSGS (*red*) and MCD (*blue*) clinical cases

In this study, we first analyzed the demographic data, basic biochemistry, and serum suPAR and uPA levels in 18 FSGS and 22 MCD patients. As shown in Table 1, FSGS patients presented higher suPAR levels compared to MCD patients (3.67 ± 0.17 vs. 2.03 ± 0.18 ng/ml; *p* < 0.05), but significant lower levels of the uPA (0.1 ± 0.02 vs. 0.44 ± 0.15 ng/ml; *p* < 0.05). In addition, human serum suPAR levels showed a moderate correlation to serum Cr levels in FSGS patients (r^2^ = 0.584, *p < 0.0001*) (Fig. 1). There were no significant differences in plasma levels of albumin or Up/Ucr between FSGS and MCD patients. The data imply that increased suPAR and decreased uPA levels could be indicators for FSGS, and there may be important functions of uPA and suPAR in FSGS.

**Table 1:** Biochemical and serologic features of clinical subjects

Subject	Age (years)	Gender	suPAR (ng/ml)	uPA (ng/ml)	Creatinine (mg/dl)	U_p_/U_Cr_
MCD	36.00 ± 4.25	3 F/19 M	2.03 ± 0.18	0.44 ± 0.15	1.13 ± 0.26	7.7 ± 0.03
FSGS	56.83 ± 8.29	4 F/14 M	3.67 ± 0.17*	0.1 ± 0.02*	3.42 ± 1.41*	8.92 ± 4.12

*uPA* urokinase-type plasminogen activator, *suPAR* soluble uPA receptor. * *p*<0.05 vs. MCD

### Deficiency of the uPA accelerated the progression of FSGS in a mice model

**Fig. 2:**
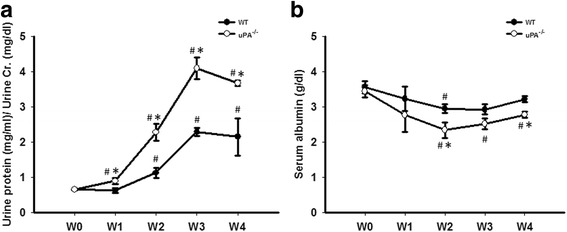
Deficiency of the urokinase-type plasminogen activator (uPA) accelerated the decrease of renal functions in a focal segmental glomerulosclerosis (FSGS) mouse model. **a** Proteinuria was expressed as total protein (mg/ml) over creatinine (mg/dl), and (**b**) plasma albumin was determined in wild-type (WT) and uPA^−/−^ groups during the course of the experiment until 4 weeks after Adriamycin treatment. ^#^ *p* < 0.05 vs. week 0 (W0); *p < 0.05 vs. the WT

**Fig. 3:**
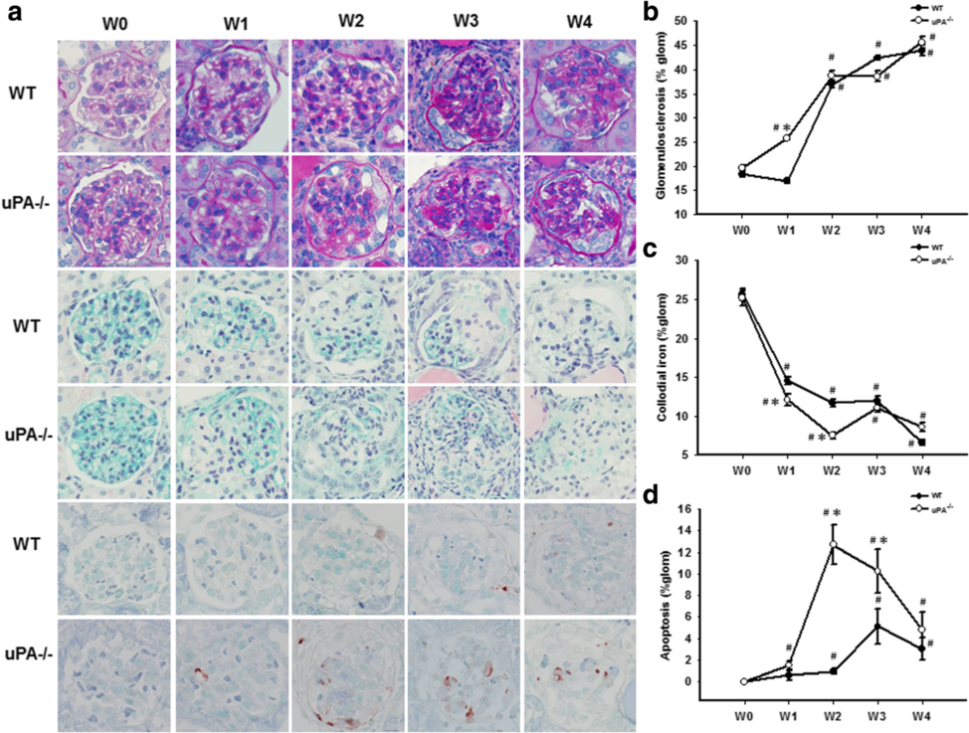
Deficiency of the urokinase-type plasminogen activator (uPA) exacerbated the severity of renal histopathological changes in a focal segmental glomerulosclerosis (FSGS) mouse model. At the end of the experiment, kidney samples were collected for a histopathological examination. **a** Representative sections from renal tissue in wild-type (WT) and uPA^−/−^ groups were evaluated with PAS staining (*lines 1 and 2*), colloidal iron staining (*lines 3 and 4*), and TUNEL staining (*lines 5 and 6*), at an original magnification of 400x. Semi-quantification analyses of PAS (**b**), colloidal iron (**c**), and TUNEL (**d**) staining are also presented. ^#^ *p* < 0.05 vs. week 0 (W0); *p < 0.05 vs. the WT

To elucidate the role of the uPA in FSGS, we used uPA^−/−^ mice in an established FSGS mice model. FSGS in this mice model was induced by an intravenous injection of Adriamycin, and mice exhibited proteinuria and focal sclerosis of glomeruli, which are similar to early pathological features in FSGS human subjects. Compared to the WT group, the uPA^−/−^ group showed significant decreases in renal functions, with earlier onset of worsening proteinuria and hypoalbuminemia and faster deterioration (Fig. 2). As for PAS staining of kidney tissues, the degree of glomerulosclerosis increased with progression of the disease in both groups, and the severity in uPA^−/−^ mice was significantly higher than that in WT mice at W1 (Fig. 3a, b). In the earlier stage of the progression (W1 and W2), expression of glomerular polyanions in the uPA^−/−^ group was also significantly lower compared to that of the WT group (Fig. 3c). In addition, uPA^−/−^ mice also showed an increase in glomerular cell apoptosis at W2 and W3 (Fig. 3d). This evidence indicates that in addition to exacerbated renal dysfunction, uPA^−/−^ mice showed accelerated destruction of the glomerular structural integrity due to increased glomerular cell apoptosis.

**Fig. 4:**
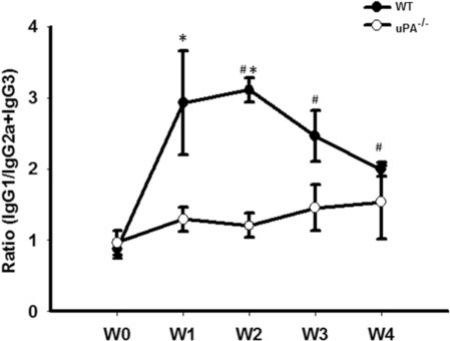
Urokinase-type plasminogen activator (uPA)-deficient mice failed to generate a type 2 immune response in a focal segmental glomerulosclerosis (FSGS) mouse model. Plasma samples in the wild-type (WT) and uPA^−/−^ groups were collected at indicated time points to determine levels of immunoglobulin G1 (IgG1), IgG2a, and IgG3. Data are presented as the ratio of IgG1 over the combination of IgG2a and IgG3. ^#^ *p* < 0.05 vs. week 0 (W0); *p < 0.05 vs. the WT

Differential subsets of helper T cells were proposed to be involved in the pathogenesis of glomerular nephritis. Th1-predominant immune responses are strongly associated with proliferative and crescentic forms of GN, while Th2 responses are associated with membranous patterns of injury [27]. Th1 and Th2 cells can antagonize each other’s actions, suggesting that certain strategies in modulating the Th1/Th2 balance may influence the disease severity. The Th1/Th2 balance takes into consideration both Th1 IgG2a and IgG3 subclasses compared to the Th2 IgG1 subclass. The ratio of IgG1/IgG2a + IgG3 was significantly increased in the WT group from W1, but there was no change in the uPA^−/−^ group (Fig. 4), suggesting that uPA^−/−^ mice fail to generate a type 2 immune response which may contribute to acceleration of Adriamycin-induced FSGS.

### Both the intact and cleaved forms of the suPAR were higher in FSGS, and the uPA, elastase, and cathepsin G were not involved in the cleavage process

**Fig. 5:**
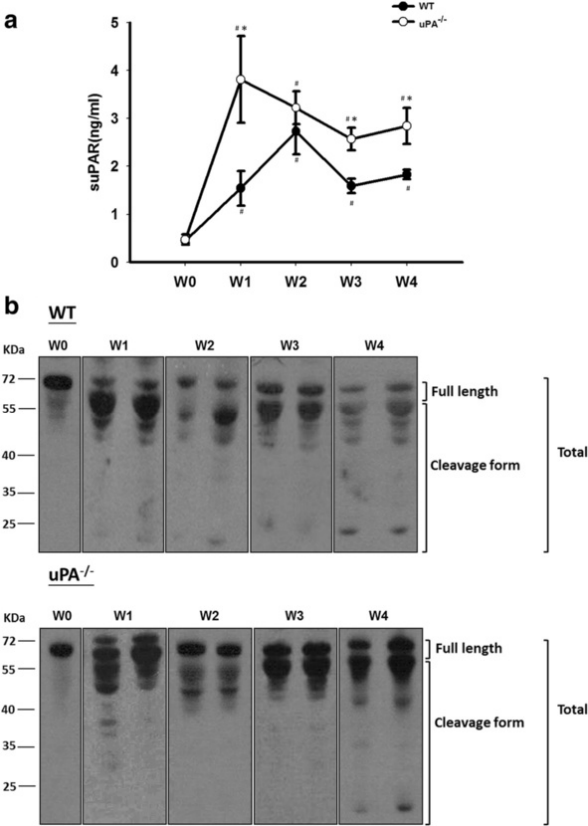
Plasma soluble urokinase-type plasminogen activator (uPA) receptor (suPAR) levels and expression patterns in a focal segmental glomerulosclerosis (FSGS) mouse model. **a** Plasma suPAR levels were determined in the wild-type (WT) and uPA^−/−^ groups during the course of the experiment until 4 weeks after Adriamycin treatment. **b** Immunoblot analysis of the expression of plasma suPAR variants in the WT (*upper panel*) and uPA^−/−^ (*lower panel*) groups at the indicated time points are presented. ^#^ *p* < 0.05 vs. week 0 (W0); *p < 0.05 vs. the WT

**Fig. 6:**
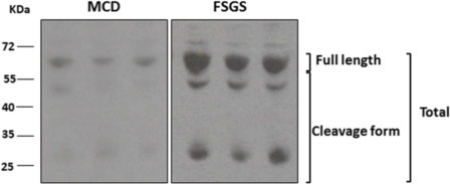
Expression patterns of soluble urokinase-type plasminogen activator receptor (suPAR) variants in clinical subjects. Immunoblot analyses of expressions of serum suPAR variants in minimal-change disease (MCD) and focal segmental glomerulosclerosis (FSGS) clinical subjects are presented

As mentioned earlier, there may be an interaction between uPA and suPAR levels in the progression of FSGS. Before the induction of FSGS, there was no difference in plasma suPAR levels between the WT and uPA^−/−^ groups. In the FSGS model, suPAR levels gradually increased after induction and reached the highest level at W2 in the WT group, while uPA^−/−^ mice presented the highest suPAR levels at W1. In addition, compared to the WT group, plasma suPAR levels all increased at different time points in the uPA^−/−^ group (Fig. 5a). The anti-uPAR antibody used herein was generated by Leu24­Thr297 of the uPAR, and therefore can be applied to discriminate the intact form(s) of the suPAR (D1D2D3_1–277_) and cleaved form(s) (D2D3_84–274_) by a Western blot analysis. As shown in Fig. 5b, two different groups of detected bands were noted: one was around 55 kDa, the other was <55 kDa, which are respectively denoted as the intact and cleaved forms. The presence of cleaved suPAR forms increased in both groups compared to levels before induction. However, there was no significant difference between the WT and uPA^−/−^ groups, suggesting that cleavage of the suPAR is independent of the uPA. In addition, we further examined the composition of intact and cleaved forms of the suPAR in clinical subjects, and an increase in the cleaved-form of the suPAR was also found in FSGS (Fig. 6). According to the data, the cleaved forms increased in the FSGS animal model and clinical subjects, suggesting that the increase in cleaved forms of the suPAR may also play a role in FSGS.

**Fig. 7:**
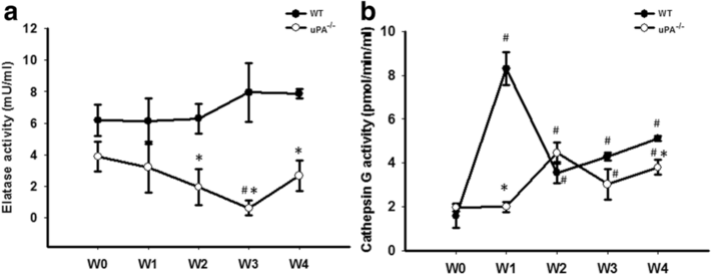
Neither elastase nor cathepsin G mediated cleavage of the soluble urokinase-type plasminogen activator (uPA) receptor (suPAR) in a focal segmental glomerulosclerosis (FSGS) mouse model. Plasma activities of elastase (**a**) and cathepsin G (**b**) were determined in the wild-type (WT) and uPA^−/−^ groups during the course of the experiment until 4 weeks after Adriamycin treatment. ^#^ *p* < 0.05 vs. week 0 (W0); *p < 0.05 vs. the WT

It was reported that the secretable cationic neutral serine proteases, elastase and cathepsin G, can cleave the uPAR and regulate its functions [28]. We further examined the activities of these two proteinases in the FSGS mice model. In the progression of FSGS, elastase activity had increased at W3, while cathepsin G activity reached its highest level at W1 (Fig. 7). However, activities of both elastase and cathepsin G in the uPA^−/−^ group were lower than those in the WT group, indicating that these two proteases did not participate in cleaving the suPAR in FSGS.

## Discussion

The meaning of circulating suPAR levels in FSGS is still under debate. In two different ethnic cohorts of primary FSGS, serum suPAR levels were elevated in 84.3 and 55.3 % of FSGS patients, while only in about 6 % of control subjects. Treatment with mycophenolate mofetil and reduction of proteinuria were both associated with a reduction in suPAR, and gave higher odds for complete remission in a clinical trial study [3]. Furthermore, evidence has been reported that NPHS2 mutations were detected in 45 % ~ 55 % of families and 8 % ~ 20 % of patients with sporadic nephrotic syndrome [29]. FSGS patients with an NPHS2 mutation also presented higher suPAR levels than those without a mutation [3]. However, recent studies suggested that eGFR is a potent determinant of suPAR levels [4, 30] and that an absolute, eGFR-independent, suPAR concentration cutoff (3.0 ng/ml) could not successfully be determined for FSGS patients [4]. In addition, Sinha et al. reported that suPAR levels could not identify FSGS among other causes of nephrotic syndrome in children [30]. In another study, suPAR levels were increased, but researchers were unable to distinguish primary from secondary FSGS [31]. In the current study, FSGS patients had higher suPAR levels (3.67 ± 0.17 ng/ml) compared to MCD patients (2.13 ± 0.18 ng/ml) and serum suPAR levels showed a moderate correlation to serum Cr levels in FSGS patients. Furthermore, suPAR levels were significantly higher at W1 after Adriamycin treatment and stayed elevated throughout the study. The uPA-deficient mice model showed higher suPAR levels and faster disease progression than WT mice. These data indicate that suPAR levels can be used to evaluate the disease in both clinical subjects and an animal model of FSGS.

It is of note that, in addition to FSGS, increased concentration of suPAR has been observed in different glomerular diseases. In a recent study, we also found serum suPAR was elevated in most kidney diseases, such as diabetic nephropathy [32], but not MCD. Zhao et al. also reported plasma suPAR levels of patients with IgA nephropathy (IgAN) were significantly lower than those in patients with primary FSGS, and higher than those with MN, MCD and healthy controls. In IgAN patients, plasma suPAR levels were positively associated with proteinuria and negatively associated with eGFR [33]. Although serum suPAR levels had some diagnostic value in FSGS, a more precise determinant cut-off value and suPAR combinations with other biomarkers are still needed.

Although use of suPAR as a diagnosis tool is under debate, it is certainly a factor in the pathogenesis of FSGS. The current methodology of determining suPAR levels might not be able to reliably distinguish FSGS from other proteinuric glomerular diseases [34]. It has been suggested that variants of the suPAR may also have diagnostic and prognostic values [22], which may be another indicator for an FSGS diagnosis. Herein, we performed a WB analysis to differentiate the suPAR variants in both animal models and clinical subjects. As shown in Fig. 5, the cleaved form increased in the FSGS animal model after induction. Both the intact and cleaved forms increased in clinical subjects, but this process was not mediated by uPA, elastase, or cathepsin G. Wei et al., reported circulating suPAR regulated kidney permeability via β3 integrin down-stream signaling in podocytes, promoting cell motility and activation of the small GTPases Cdc42 and Rac1 [35]. However, which fractions of suPAR mediated the activation of β3 integrin remains unclear. In a recently study, Alfano et al., provided in vitro evidence that full-length suPAR but not cleaved suPAR variant has the capability to induce nephrin down-regulation in human podocytes [36]. The detailed mechanisms and functions of increased cleavage in suPAR variants in FSGS still need further investigation. Although it is still too early to draw conclusions about suPAR variants in an FSGS diagnosis, we hope to have additional information after further studies. In addition to serum suPAR levels for an FSGS diagnosis, it was reported that the urinary suPAR is elevated and pathogenic in patients with primary FSGS [37] and may be used to identify cases of recurrent FSGS in kidney transplant candidates [38].

Expansion of the extracellular matrix (ECM) might not only result from increased matrix protein synthesis by fibroblasts but also from decreased degradation by proteases such as metalloproteinases and serine proteases. Actually, uPA is considered to be a logical source of endogenous renal antifibrotic activity through enhancing the process of protein degradation [39]. In addition, uPA has been reported to have the ability to promote tissue repair through activation of growth factors, such as hepatocyte growth factor (HGF) [40, 41]. Stimulation of podocytes with HGF in vitro resulted in antiapoptotic phosphorylation of AKT and extracellular signal-regulated kinase and induction of the X-linked inhibitor of apoptosis protein. In the same study, diminution of the HGF downstream signaling pathway in podocytes contributes to cell loss and FSGS in transplant glomerulopathy [42]. However, evidence from uPA and uPAR deficient animals has shown other molecules, such as vitronectin and high molecular weight kininogen, are alternate uPAR ligands, and receptors in addition to uPAR may also bind directly to uPA and activate cell signaling pathways [43]. In a renal interstitial fibrosis obstructive nephropathy model (UUO) [44], uPAR deficiency suppressed renal macrophage recruitment but exacerbated the fibrogenic response, which may partly have occurred through the delayed clearance of angiogenic/profibrotic molecules and decreased receptor-associated uPA activity. In addition, the number of apoptotic tubulointerstitial cells was also significantly increased in uPAR deficient mice [45]. In contrast, Yamaguchi I et al., found uPA deficiency had no role in the inhibition of interstitial fibrosis in the UUO model, and suggested an organ-specific difference in basic fibrogenic pathways [39]. Treatment with recombinant suPAR to uPAR-knockout mice with 20 μg and greater led to albuminuria within 24 h. In addition, sustained overexpression of suPAR in the blood of WT mice leads to an FSGS-like glomerulopathy [2]. Here, we used an Adriamycin-induced FSGS model in uPA- knockout mice to elucidate the role of uPA in FSGS. In the mice model of our current study, uPA deficient accelerated Adriamycin-induced FSGS in the early stage, but did not increase the severity in the late stage (Fig. 3). This evidence suggests uPA may play only a partial role in ameliorating ECM expansion in the initial stage of FSGS.

It has been claimed that the pathogenesis of FSGS is mainly mediated by the podocyte damage and loss. This pathological process includes dysregulated slit diaphragm, cytoskeleton, glomerular basement membrane, and affected negative surface charge of the podocyte. An injured podocyte will trigger cell apoptosis and detachment from glomerular basement membrane, loss of glomerular structural integrity and result in sclerosis and scarring of the glomerulus [46]. In the current study, we observed that the increase of glomerular apoptotic cells and lower glomerular polyanions in uPA- deficient mice might imply an anti-apoptotic role of uPA in the pathogenesis of FSGS (Fig. 3). The underlying mechanisms may be mediated through the activation of caspase-mediated apoptosis and inhibit the PI3K/AKT pathway [47]. In addition, uPA has been suggested as a major determinant of the basal level of activated ERK/MAP kinase, and may prevent cell apoptosis [48]. However, it has been reported that uPA increases survival or pro-apoptotic signals in human mesangial cells depending on the apoptotic stimulus [49]. The detailed mechanisms of uPA anti-apoptotic activity still need further investigation.

The immunological mechanisms involved in the pathogenesis of FSGS are unclear, but evidence suggests an association between Th2-polarization and disease development [50]. For example, Yap et al. reported a correlation between childhood idiopathic nephrotic syndrome and increased IL-13 mRNA expression. In Buffalo/Mna spontaneously developed FSGS rat model, a predominantly Th2 mRNA profile and the down-regulation of Th1 cytokine mRNA was also observed [51]. It is known Th1 and Th2 cells can provide B-cell help, and their signature cytokines IFN-γ and IL-4 induce class switch recombination to IgG2a and IgG3 or IgG1 and IgE, respectively [52]. In our study, uPA-deficient animals didn’t switch to IgG2a and IgG3, but the progression of FSGS was still accelerated. This indicates the role of Th2 polarization maybe less important than a uPA deficiency in the mice model of FSGS induced by Adriamycin. At present, we are the first group to present the idea and possible hypothesis of the role of Th2 polarization in the development of FSGS which may be only applicable in certain models or conditions and not a general effect.

## Conclusion

In summary, we found that the increase in suPAR and decrease in uPA levels both occurred in FSGS clinical subjects. A gradual increase in suPAR levels was also present in a mouse model of Adriamycin-induced FSGS. In addition, disruption of uPA accelerated the onset of FSGS and exacerbated its severity in proteinuria and cell apoptosis. Dysregulation of the Th1/Th2 balance may also be involved in uPA-regulated disease progression. In addition, the suPAR in FSGS animals was manifested in the cleaved form, while it was predominantly in the intact form in control animals, and the cleavage process was not mediated by the uPA, elastase, or cathepsin G through an enzymatic process. These data suggest the levels of serum uPA may be an indicator for the progression of FSGS in clinical subjects and animal models.

## References

[1] McCarthy ET, Swan SK, Ellis E, Savin VJ, Sharma R, Sharma M (1996). Circulating factor associated with increased glomerular permeability to albumin in recurrent focal segmental glomerulosclerosis. N Engl J Med.

[2] Fornoni A, Goes N, Sageshima J, Wei C, El Hindi S, Li J (2011). Circulating urokinase receptor as a cause of focal segmental glomerulosclerosis. Nat Med.

[3] Dong C, Friedman AL, Gassman JJ, Wei C, Trachtman H, Li J (2012). Circulating suPAR in two cohorts of primary FSGS. J Am Soc Nephrol.

[4] Bammens B, Poesen R, Claes K, Meijers B, Maas RJ, Sprangers B (2014). The soluble urokinase receptor is not a clinical marker for focal segmental glomerulosclerosis. Kidney Int.

[5] Maas RJ, Deegens JK, Wetzels JF (2013). Serum suPAR in patients with FSGS: trash or treasure?. Pediatr Nephrol.

[6] Blasi F, Vassalli JD, Dano K (1987). Urokinase-type plasminogen activator: proenzyme, receptor, and inhibitors. J Cell Biol.

[7] Thuno M, Macho B, Eugen-Olsen J (2009). suPAR: the molecular crystal ball. Dis Markers.

[8] Gerard CJ, Rosenberg S, Chapman HA, Waltz DA, Fujita RM, Yang X (2000). Nonproteolytic role for the urokinase receptor in cellular migration in vivo. Am J Respir Cell Mol Biol.

[9] Bender JR, Blasi F, Pardi R, Bianchi E, Ferrero E, Fazioli F (1996). Integrin-dependent induction of functional urokinase receptors in primary T lymphocytes. J Clin Invest.

[10] Dinh DH, Gujrati M, Rao JS, Raghu H, Lakka SS, Gondi CS (2010). Suppression of uPA and uPAR attenuates angiogenin mediated angiogenesis in endothelial and glioblastoma cell lines. PLoS One.

[11] Ploug M, Ellis V (1994). Structure-function relationships in the receptor for urokinase-type plasminogen activator. Comparison to other members of the Ly-6 family and snake venom alpha-neurotoxins. FEBS Lett.

[12] Jensen AL, Blasi F, Danø K, Ploug M, Ronne E, Behrendt N (1991). Cellular receptor for urokinase plasminogen activator. Carboxyl-terminal processing and membrane anchoring by glycosyl-phosphatidylinositol. J Biol Chem.

[13] Gilquin B, Stura EA, Ménez A, Llinas P, Le Du MH, Gardsvoll H (2005). Crystal structure of the human urokinase plasminogen activator receptor bound to an antagonist peptide. EMBO J.

[14] Furie BC, Cines DB, Huang M, Huai Q, Mazar AP, Kuo A (2006). Structure of human urokinase plasminogen activator in complex with its receptor. Science.

[15] Blasi F, Carmeliet P (2002). uPAR: a versatile signalling orchestrator. Nat Rev Mol Cell Biol.

[16] Hoyer-Hansen G, Lund IK (2007). Urokinase receptor variants in tissue and body fluids. Adv Clin Chem.

[17] Weidle U, Danø K, Behrendt N, Hoyer-Hansen G, Pessara U, Holm A (2001). Urokinase-catalysed cleavage of the urokinase receptor requires an intact glycolipid anchor. Biochem J.

[18] Lund LR, Ellis V, Danø K, Hoyer-Hansen G, Ronne E, Solberg H (1992). Urokinase plasminogen activator cleaves its cell surface receptor releasing the ligand-binding domain. J Biol Chem.

[19] Elonen E, Alitalo R, Vaheri A, Mustjoki S, Sidenius N, Sier CF (2000). Soluble urokinase receptor levels correlate with number of circulating tumor cells in acute myeloid leukemia and decrease rapidly during chemotherapy. Cancer Res.

[20] Dybkjaer E, Danø K, Brünner N, Stephens RW, Pedersen AN, Nielsen HJ (1997). ELISA determination of soluble urokinase receptor in blood from healthy donors and cancer patients. Clin Chem.

[21] Slot O, Brunner N, Locht H (1999). Soluble urokinase plasminogen activator receptor in plasma of patients with inflammatory rheumatic disorders: increased concentrations in rheumatoid arthritis. Ann Rheum Dis.

[22] Rasch MG, Lund IK, Almasi CE, Hoyer-Hansen G (2008). Intact and cleaved uPAR forms: diagnostic and prognostic value in cancer. Front Biosci.

[23] Chen A, Sytwu HK, Lin YF, Wu CC, Chen JS, Lin SH (2008). Experimental model of membranous nephropathy in mice: sequence of histological and biochemical events. Lab Anim.

[24] Ljubanović D, Holers VM, Thurman JM, Strassheim D, Renner B, Panzer S (2013). IgM contributes to glomerular injury in FSGS. J Am Soc Nephrol.

[25] Donati S, Orefici G, Nisini R, Teloni R, von Hunolstein C, Mariotti S (2004). Antibody classes & subclasses induced by mucosal immunization of mice with Streptococcus pyogenes M6 protein & oligodeoxynucleotides containing CpG motifs. Indian J Med Res.

[26] Altshuler AE, Penn AH, Yang JA, Kim GR, Schmid-Schönbein GW (2012). Protease activity increases in plasma, peritoneal fluid, and vital organs after hemorrhagic shock in rats. PLoS One..

[27] Cheng CW, Chang WL, Chang LC, Wu CC, Lin YF, Chen JS. Ferulic Acid, an Angelica sinensis-Derived Polyphenol, Slows the Progression of Membranous Nephropathy in a Mouse Model. Evid Based Complement Alternat Med 2012; 2012: 161235 doi:10.1155/2012/161235. 10.1155/2012/161235 PMC3403610 22844329

[28] Namane A, Chignard M, Pidard D, Beaufort N, Leduc D, Rousselle JC (2004). Proteolytic regulation of the urokinase receptor/CD87 on monocytic cells by neutrophil elastase and cathepsin G. J Immunol.

[29] Caridi G, Perfumo F, Ghiggeri GM (2005). NPHS2 (Podocin) mutations in nephrotic syndrome. Clinical spectrum and fine mechanisms. Pediatr Res.

[30] Bagga A, Hari P, Pandey RM, Sinha A, Bajpai J, Saini S (2014). Serum-soluble urokinase receptor levels do not distinguish focal segmental glomerulosclerosis from other causes of nephrotic syndrome in children. Kidney Int.

[31] Chu R, Chen Y, Zhao MH, Huang J, Liu G, Zhang YM (2013). Plasma soluble urokinase receptor levels are increased but do not distinguish primary from secondary focal segmental glomerulosclerosis. Kidney Int.

[32] Chen JS, Chu NF, Pei D, Wu CZ, Chang LC, Lin YF (2015). Urokinase plasminogen activator receptor and its soluble form in common biopsy-proven kidney diseases and in staging of diabetic nephropathy. Clin Biochem.

[33] Zhang H, Zhao M, Liu G, Zhao Y, Liu L, Huang J (2015). Plasma soluble urokinase receptor level is correlated with podocytes damage in patients with IgA nephropathy. PLoS One.

[34] Schlondorff D (2014). Are serum suPAR determinations by current ELISA methodology reliable diagnostic biomarkers for FSGS?. Kidney Int.

[35] Carmeliet P, Mundel P, Reiser J, Wei C, Moller CC, Altintas MM (2008). Modification of kidney barrier function by the urokinase receptor. Nat Med.

[36] Mikulak J, Mavilio D, Saleem MA, Alfano M, Cinque P, Giusti G (2015). Full-length soluble urokinase plasminogen activator receptor down-modulates nephrin expression in podocytes. Sci Rep.

[37] Zhao MH, Chu R, Liu XJ, Huang J, Liu G, Zhang YM (2014). Urinary soluble urokinase receptor levels are elevated and pathogenic in patients with primary focal segmental glomerulosclerosis. BMC Med.

[38] Stegall MD, Cosio FG, Amer H, Franco Palacios CR, Lieske JC, Wadei HM (2013). Urine but not serum soluble urokinase receptor (suPAR) may identify cases of recurrent FSGS in kidney transplant candidates. Transplantation.

[39] Collins SJ, Okamura DM, Eddy AA, Yamaguchi I, Lopez-Guisa JM, Cai X (2007). Endogenous urokinase lacks antifibrotic activity during progressive renal injury. Am J Physiol Renal Physiol.

[40] Novak ML, Simon RH, Koh TJ, Sisson TH, Nguyen MH, Yu B (2009). Urokinase-type plasminogen activator increases hepatocyte growth factor activity required for skeletal muscle regeneration. Blood.

[41] Tsubouchi H, Blasi F, Comoglio PM, Naldini L, Tamagnone L, Vigna E (1992). Extracellular proteolytic cleavage by urokinase is required for activation of hepatocyte growth factor/scatter factor. EMBO J.

[42] Kreipe HH, Bröcker V, Becker JU, Agustian PA, Schiffer M, Gwinner W (2011). Diminished met signaling in podocytes contributes to the development of podocytopenia in transplant glomerulopathy. Am J Pathol.

[43] Zhang G, Eddy AA (2008). Urokinase and its receptors in chronic kidney disease. Front Biosci.

[44] Chevalier RL, Forbes MS, Thornhill BA (2009). Ureteral obstruction as a model of renal interstitial fibrosis and obstructive nephropathy. Kidney Int.

[45] Lopez-Guisa JM, Carmeliet P, Eddy AA, Zhang G, Kim H, Cai X (2003). Urokinase receptor modulates cellular and angiogenic responses in obstructive nephropathy. J Am Soc Nephrol.

[46] Reidy K, Kaskel FJ (2007). Pathophysiology of focal segmental glomerulosclerosis. Pediatr Nephrol.

[47] Gondi CS, Kandhukuri N, Dinh DH, Gujrati M, Rao JS (2007). Down-regulation of uPAR and uPA activates caspase-mediated apoptosis and inhibits the PI3K/AKT pathway. Int J Oncol..

[48] Ma Z, Webb DJ, Jo M, Gonias SL (2001). Endogenously produced urokinase-type plasminogen activator is a major determinant of the basal level of activated ERK/MAP kinase and prevents apoptosis in MDA-MB-231 breast cancer cells. J Cell Sci.

[49] Shushakova N, Haller H, Dumler I, Tkachuk N, Kiyan J, Tkachuk S (2008). Urokinase induces survival or pro-apoptotic signals in human mesangial cells depending on the apoptotic stimulus. Biochem J.

[50] Savage PB, Câmara NO, Keller AC, Pereira RL, Reis VO, Semedo P (2012). Invariant natural killer T cell agonist modulates experimental focal and segmental glomerulosclerosis. PLoS One.

[51] Sim SK, Seah CC, Jordan SC, Yap HK, Cheung W, Murugasu B (1999). Th1 and Th2 cytokine mRNA profiles in childhood nephrotic syndrome: evidence for increased IL-13 mRNA expression in relapse. J Am Soc Nephrol.

[52] Taylor DR, MacLennan IC, Acha-Orbea H, Toellner KM, Luther SA, Sze DM (1998). T helper 1 (Th1) and Th2 characteristics start to develop during T cell priming and are associated with an immediate ability to induce immunoglobulin class switching. J Exp Med.

