# Erratum to: Significance of the urokinase-type plasminogen activator and its receptor in the progression of focal segmental glomerulosclerosis in clinical and mouse models

**DOI:** 10.1186/s12929-017-0348-6

**Published:** 2017-07-19

**Authors:** Jin-Shuen Chen, Li-Chien Chang, Chung-Ze Wu, Tzu-Ling Tseng, Jui-An Lin, Yuh-Feng Lin, Chao-Wen Cheng

**Affiliations:** 1 Division of Nephrology, Department of Internal Medicine, Tri-Service General Hospital, National Defense Medical Center, No. 325, Section 2, Chenggong Road, Neihu District, Taipei, 114 Taiwan; 2 School of Pharmacy, National Defense Medical Center, Taipei, Taiwan; 3 Department of Internal Medicine, Shuang Ho Hospital, Taipei Medical University, New Taipei City, Taiwan; 4 Biomedical Technology & Device Research Laboratories, Industrial Technology Research Institute, Hsinchu, Taiwan; 5 Graduate Institute of Clinical Medicine, College of Medicine, Taipei Medical University, No. 250, Wu-Xing Street, Taipei, 110 Taiwan; 6 Graduate Institute of Medical Sciences, National Defense Medical Center, Taipei, Taiwan

## Erratum

Following publication of this article [1] it has come to our attention that one of the supported grants from the Tri-Service General Hospital was omitted:

“We thank all subjects who participated in the study. This work was supported by grants from the National Defense Medical Center, the Tri-Service General Hospital (**TSGH-C103-007-S02**), and the Ministry of Science and Technology of Taiwan (MOST 102-2314-B-016-012-), Taiwan. This work was also partly supported by a grant from Taipei Medical University (TMU100-AE3-Y06). The authors thank Ms. Hsin-Yi Tsai for her technical support”.

This should have been presented as: “We thank all subjects who participated in the study. This work was supported by grants from the National Defense Medical Center, the Tri-Service General Hospital (**TSGH-C103-007-S02, TSGH-C104-007-007-S02**), and the Ministry of Science and Technology of Taiwan (MOST 102-2314-B-016-012-), Taiwan. This work was also partly supported by a grant from Taipei Medical University (TMU100-AE3-Y06). The authors thank Ms. Hsin-Yi Tsai for her technical support”.

The authors would like to sincerely apologize for any inconvenience or confusion this may have caused.
